# Long-Term Stability of Allergic Bronchopulmonary Mycosis After Bronchoscopic Removal of Mucus Plugs: A Six-Year Follow-Up Case Report

**DOI:** 10.7759/cureus.98885

**Published:** 2025-12-10

**Authors:** Tomoyuki Araya, Toshiyuki Kita, Takayuki Higashi, Ryo Hara, Hazuki Takato

**Affiliations:** 1 Respiratory Medicine, National Hospital Organization (NHO) Kanazawa Medical Center, Kanazawa, JPN

**Keywords:** allergic asthma, allergic bronchopulmonary mycosis, flexible fiberoptic bronchoscopy (ffb), mucus plug, penicillium citrinum

## Abstract

A 37-year-old man with asthma under treatment presented with fever and dyspnea, accompanied by marked peripheral eosinophilia (10,400/µL) and elevated total immunoglobulin E (IgE) levels (463.2 IU/mL). Chest computed tomography demonstrated multiple mucus plugs in the right upper lobe and bilateral lower lobes, accompanied by central bronchiectasis. Bronchoscopy revealed mucus plugs in the tracheal bifurcation and lower bronchi. Cultures of the removed sputum and mucus plugs grew *Penicillium citrinum*. Pathology of the mucus plugs showed necrotic and degenerated cells, exudates, mucus, and Charcot-Leyden crystals. Based on these clinical, serological, and radiological findings, the diagnostic criteria for allergic bronchopulmonary mycosis (ABPM) were fulfilled. Following bronchoscopic removal of the mucus plugs led to rapid improvement without pharmacological therapy. No recurrence was observed for more than six years. This rare case suggests that bronchoscopic mucus plug removal may serve not only for diagnosis but also as an effective therapeutic option in selected patients with ABPM caused by *Penicillium citrinum*.

## Introduction

Allergic bronchopulmonary mycosis (ABPM) is a chronic inflammatory airway disorder in which saprophytic fungi colonizing the bronchi trigger type I and type III hypersensitivity reactions [1]. This immune response leads to mucus hypersecretion, airway obstruction, and, in some patients, progressive bronchiectasis. Diagnosis is typically based on the clinical criteria proposed by Asano et al. [2], incorporating asthma-like symptoms, peripheral eosinophilia, elevated serum immunoglobulin E (IgE), fungus-specific IgE/immunoglobulin G antibodies, characteristic radiologic findings, and identification of the causative organism.

While *Aspergillus* species are most commonly implicated, biopsy-based analyses such as that by Ishiguro et al. have shown that other fungi, including *Schizophyllum commune* and *Penicillium* species, can also cause ABPM [3]. Environmental *Penicillium* species are known to be widespread and possess IgE-reactive allergens, indicating that non-*Aspergillus* fungi may contribute to allergic airway disease in susceptible individuals [4].

Standard therapy for ABPM consists of systemic corticosteroids and oral azole antifungals [5]. However, several reports describe patients who improved solely after bronchoscopic removal of mucus plugs [6-10], indicating that relieving airway obstruction can be therapeutic in select cases. Here, we describe a rare case of ABPM caused by *Penicillium citrinum* in which clinical improvement was achieved after bronchoscopic mucus plug removal alone.

## Case presentation

A 37-year-old man presented with a one-month history of recurrent fever and worsening dyspnea. His past medical history was notable only for trigeminal neuralgia. He had no known allergies and no relevant family history. His smoking history amounted to 5 pack-years, and he quit smoking at age 25. He previously worked in a paper factory but had left the job one year earlier. He lived in a 30-year-old wooden house and had no pets.

One month before admission, he was evaluated at our hospital for a wet cough and dyspnea and diagnosed with bronchial asthma. Treatment with inhaled vilanterol 25 μg/fluticasone 200 μg (inhaled corticosteroid/long-acting β₂-agonist combination therapy), montelukast, and ambroxol was initiated. Despite these therapies, the recurrent fever and worsening dyspnea persisted, leading to readmission for further evaluation. 

On admission, his temperature was 38.1 °C, pulse 110 beats/minute, blood pressure 120/85 mmHg, and SpO₂ 92%-93% on room air. Physical examination revealed no wheezes or crackles.

Initial laboratory evaluation revealed marked leukocytosis, with a white blood cell count of 20,800/µL and striking eosinophilia of 50% (10,400/µL), along with an elevated C-reactive protein level of 4.13 mg/dL. Total serum IgE was modestly elevated at 463.2 IU/mL. Specific IgE demonstrated strong positivity for several allergens, while *Penicillium*-specific IgE was negative. Serum β-D-glucan was within the normal range, proteinase-3 antineutrophil cytoplasmic antibody and myeloperoxidase antineutrophil cytoplasmic antibody were negative, the FIP1L1-platelet-derived growth factor receptor alpha fusion gene was not detected, and stool examinations showed no ova or parasites, as summarized in Table 1.

**Table 1:** Laboratory findings including autoantibody profiles and allergen-specific IgE levels. This table summarizes the patient's hematologic, biochemical, immunologic, and allergen-specific IgE parameters obtained at presentation. General laboratory parameters are shown first, followed by autoimmune markers (ANCA, ANA, and FIP1L1–PDGFRA fusion gene analysis). A separate section labeled “Specific IgE” lists allergen-specific IgE levels measured using serum immunoassays. Reference ranges are shown in the rightmost column. WBC, white blood cell; RBC, red blood cell; CRP, C-reactive protein; T-Bil, total bilirubin; TP, total protein; Alb, albumin; ALP, alkaline phosphatase; AST, aspartate aminotransferase; ALT, alanine aminotransferase; GGT, γ-glutamyl transpeptidase; LDH, lactate dehydrogenase; Na, sodium; K, potassium; Cl, chloride; BUN, blood urea nitrogen; Cre, creatinine; UA, uric acid; CK, creatine kinase; Ag, antigen; PR3-ANCA, proteinase-3 antineutrophil cytoplasmic antibody; MPO-ANCA, myeloperoxidase antineutrophil cytoplasmic antibody; ANA, antinuclear antibody; FIP1L1–PDGFRA, FIP1-like-1–platelet-derived growth factor receptor α

Parameter	Value	Reference range
WBC (/µL)	20,800	4,500-9,000
Neutrophil (%)	26.0	38-74
Lymphocyte (%)	15.0	16.5-49.5
Eosinophil (%)	50.0	0-10
Monocyte (%)	7.0	5-10
Basophil (%)	2.0	0-2
RBC (×10⁴/µL)	533	414-575
Hemoglobin (g/dL)	15.9	13.0-17.1
Hematocrit (%)	46.5	39.6-50.8
Platelet (×10⁴/µL)	35.2	15-35
CRP (mg/dL)	4.13	0-0.4
T-Bil (mg/dL)	0.4	0.3-1.2
TP (g/dL)	6.3	6.7-8.3
Alb (g/dL)	3.8	4.0-5.0
ALP (U/L)	250	115-359
AST (U/L)	16	13-33
ALT (U/L)	31	8-42
GGT (IU/L)	61	10-47
LDH (U/L)	209	119-229
Na (mEq/L)	136	135-149
K (mEq/L)	4.2	3.5-4.9
Cl (mEq/L)	100	96-108
BUN (mg/dL)	10.1	8-22
Cre (mg/dL)	0.88	0.6-1.0
eGFR (mL/min/L)	79.2	60-100
UA (mg/dL)	5.4	3.6-7.0
CK (U/L)	98	45-163
Amylase (U/L)	50	35-140
BNP (pg/mL)	5.8	<18.4
β-D-glucan (pg/mL)	2.407	<11
Aspergillus Ag	Negative	Negative
Candida Ag	Negative	Negative
Cryptococcus Ag	Negative	Negative
IgE (IU/mL)	463.2	<232
PR3-ANCA (U/mL)	<1.0	<1.0
MPO-ANCA (U/mL)	<1.0	<1.0
ANA titer	<40	<40
FIP1L1-PDGFRA fusion gene	Negative	Negative
Specific IgE (UA/mL)		
Japanese cedar	53.4	<0.35
Orchard grass	4.77	<0.35
Dermatophagoides pteronyssinus	0.21	<0.35
Dermatophagoides farinae	0.25	<0.35
*Aspergillus* spp.	0.97	<0.35
*Candida* spp.	0.86	<0.35
*Alternaria* spp.	4.61	<0.35
*Penicillium* spp.	0.10	<0.35

**Figure 1:**
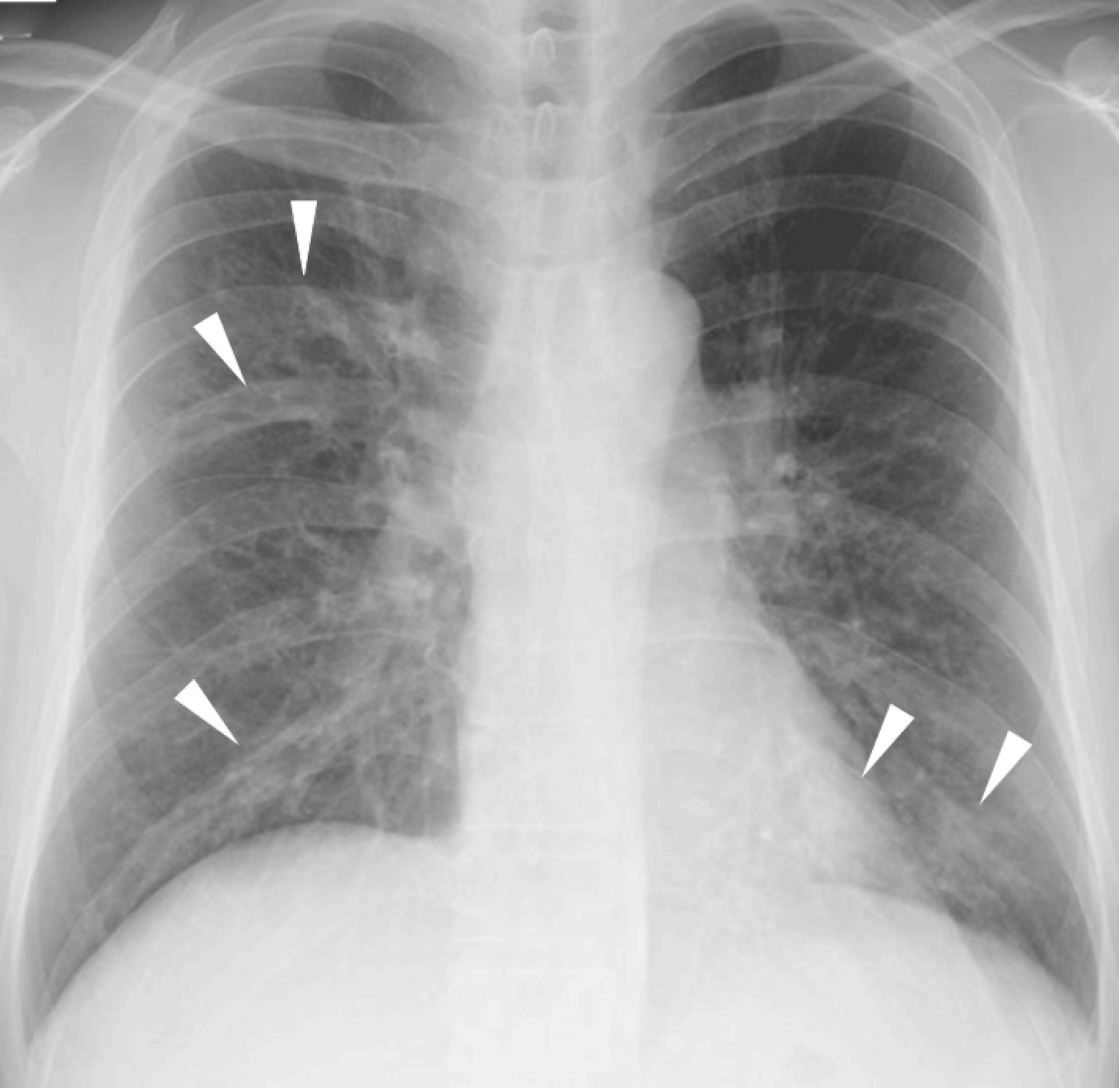
Chest X-ray demonstrating bilateral bronchovascular bundle thickening. Chest X-ray on admission shows bilateral bronchovascular bundle thickening (arrowheads), predominantly in the lower lung fields. These findings correspond to mucus plugging observed on computed tomography, reflecting airway inflammation associated with allergic bronchopulmonary mycosis.

Chest X-ray revealed bilateral bronchovascular bundle thickening, which corresponds to mucus plugging on computed tomography (CT) (Figure 1).

**Figure 2:**
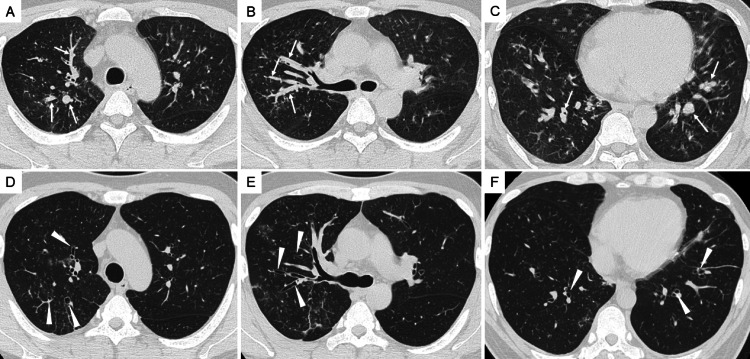
Chest CT before and after bronchoscopic mucus plug removal. (A-C) Chest CT on admission demonstrates impacted mucus plugs (arrows). Arrows in (A) and (B) indicate thick, high-attenuation mucus obstructing the right upper lobe bronchus, while the arrow in (C) highlights mucus impaction within the bilateral lower lobe bronchi, accompanied by central bronchiectasis. (D-F) Follow-up CT performed seven days after bronchoscopic mucus plug removal shows marked resolution of the previously noted mucus impaction (arrowheads) and restoration of airway patency in all affected bronchi.

Such findings are consistent with the characteristic radiological features of allergic bronchopulmonary mycosis, which typically include mucoid impaction and peribronchial thickening. Chest CT further demonstrated mucus plugs within the right upper lobe bronchus and bilateral lower lobe bronchi, accompanied by central bronchiectasis (Figures 2A-2C).

**Figure 3:**
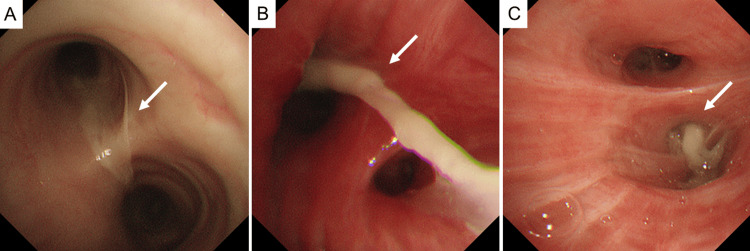
Bronchoscopic findings of mucus plug obstruction at the tracheal bifurcation and lower lobes. (A) Bronchoscopy reveals a large mucus plug partially obstructing the tracheal bifurcation (arrow). (B) In the right lower lobe bronchus, a thick, adherent mucus plug is observed, along with marked erythema and swelling of the bronchial mucosa (arrow). (C) A similar obstructing mucus plug is visualized in the left lower lobe bronchus (arrow). Multiple mucus plugs and sputum are gradually detached and aspirated as completely as possible, resulting in improved airway patency.

Bronchoscopy confirms large mucus plugs at the tracheal bifurcation and in both lower lobe bronchi, accompanied by abundant sputum. Multiple mucus plugs and sputum were aspirated and recovered as completely as possible (Figure 3).

**Figure 4:**
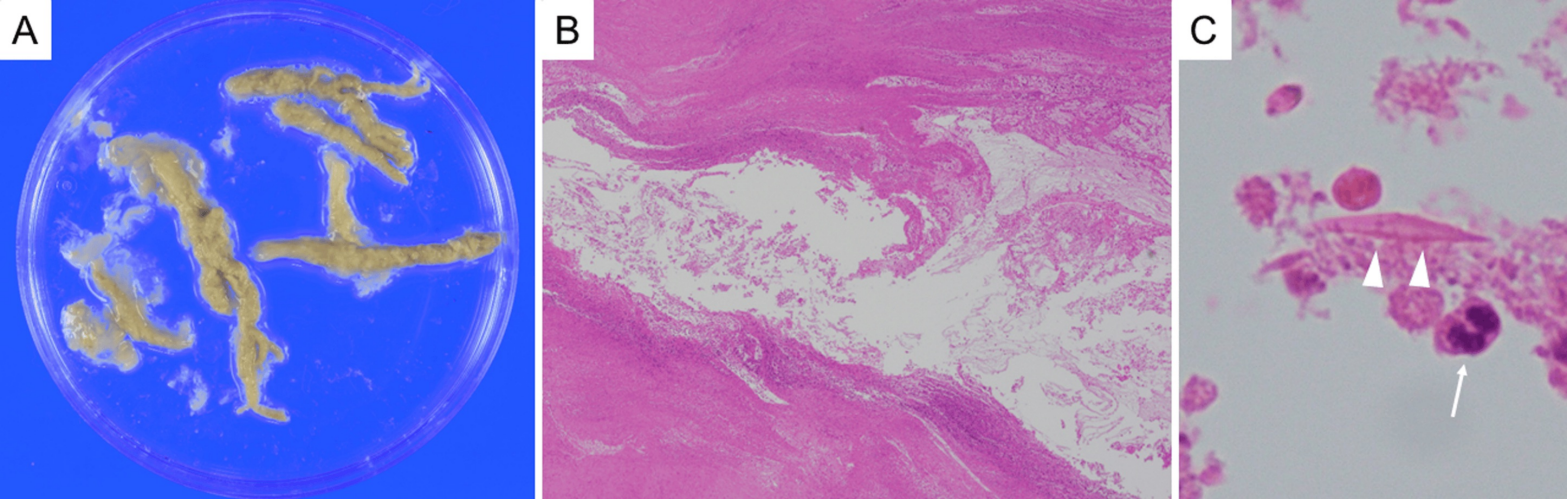
Gross and pathological characteristics of the removed mucus plugs. (A) Gross appearance of the mucus plugs removed bronchoscopically. (B) Low-magnification (×40) histopathology demonstrates necrotic and degenerated cells, exudates, and mucus. (C) High-magnification (×600) view shows Charcot-Leyden crystals (arrowheads) and eosinophils (arrow), characteristic of allergic bronchopulmonary mycosis. Periodic acid-Schiff, Grocott, and Ziehl-Neelsen stains reveal no organisms.

Plugs measuring approximately 5 mm in diameter and 30 mm in length were removed (Figure 4A).

Cultures of the removed sputum and mucus plugs grew *Penicillium citrinum*. Pathological examination of the mucus plugs showed necrotic and degenerated cells, exudates, mucus, and Charcot-Leyden crystals - features characteristic of ABPM - while Periodic acid-Schiff, Grocott, and Ziehl-Neelsen stains reveal no organisms (Figures 4B-4C).

Based on the established diagnostic criteria for allergic bronchopulmonary mycosis [2], the patient meets six key components - namely asthma-like symptoms, peripheral eosinophilia exceeding 500/µL, elevated total IgE of 417 IU/mL or higher, a positive sputum culture identifying *Penicillium citrinum*, central bronchiectasis on CT, and mucus plugs identified on CT - supporting a diagnosis of allergic bronchopulmonary mycosis caused by *Penicillium citrinum*.

Bronchoscopic mucus plug removal led to rapid improvement, with the patient experiencing relief of dyspnea and fever beginning the following day. Peripheral eosinophilia decreased steadily without systemic corticosteroids or antifungal therapy. Seven days after the procedure, chest CT demonstrated marked resolution of mucus plugging (Figures 2D-2F), and the eosinophil count had fallen to 2,760/µL. By three weeks, eosinophils had further declined to 864/µL and continued to decrease thereafter, normalizing to 158/µL (normal range: <500/µL) at nine months. Total IgE also normalized to 280.4 IU/mL (normal range: <232 IU/mL) at four months (Table 2). In addition, pulmonary function parameters showed measurable improvement by day 7 despite residual obstructive impairment, and fractional exhaled nitric oxide demonstrated a marked decline, as reflected in the longitudinal data presented in Table 2.

**Table 2:** Serial changes in eosinophils, IgE levels, lung function, and FeNO from admission through post-bronchoscopy follow-up. Time points are reported after bronchoscopy unless otherwise indicated. Eosinophil levels demonstrated a marked and progressive decline following bronchoscopic removal of mucus plugs, decreasing from 10,400/µL at admission to 2,760/µL at seven days, further to 864/µL at three weeks, and ultimately normalizing to 158/µL by nine months (reference range: <500/µL). IgE levels showed a transient and mild increase at week 3 but nearly normalized by four months. Pulmonary function parameters improved by day 7, although mild obstructive impairment persisted thereafter. FeNO values exhibited a dramatic reduction before and after bronchoscopy, reflecting rapid attenuation of eosinophilic airway inflammation. FVC, forced vital capacity; FEV1, forced expiratory volume in one second; FeNO, fractional exhaled nitric oxide

Time point	Eosinophils (/µL, <500)	IgE (IU/mL, <232)	FVC (L)	%FVC (%)	FEV1 (L)	%FEV1 (%)	FEV1/FVC (%)	FeNO (ppb, <25)
Admission (baseline)	10,400	463.2	2.20	53.5	0.94	24.7	42.7	156
Day 7	2,760	-	3.94	94.9	2.14	55.6	54.3	36
Week 3	864	590.9	-	-	-	-	-	-
Month 4	759	280.4	-	-	-	-	-	-
Month 9	158	-	-	-	-	-	-	-

**Figure 5:**
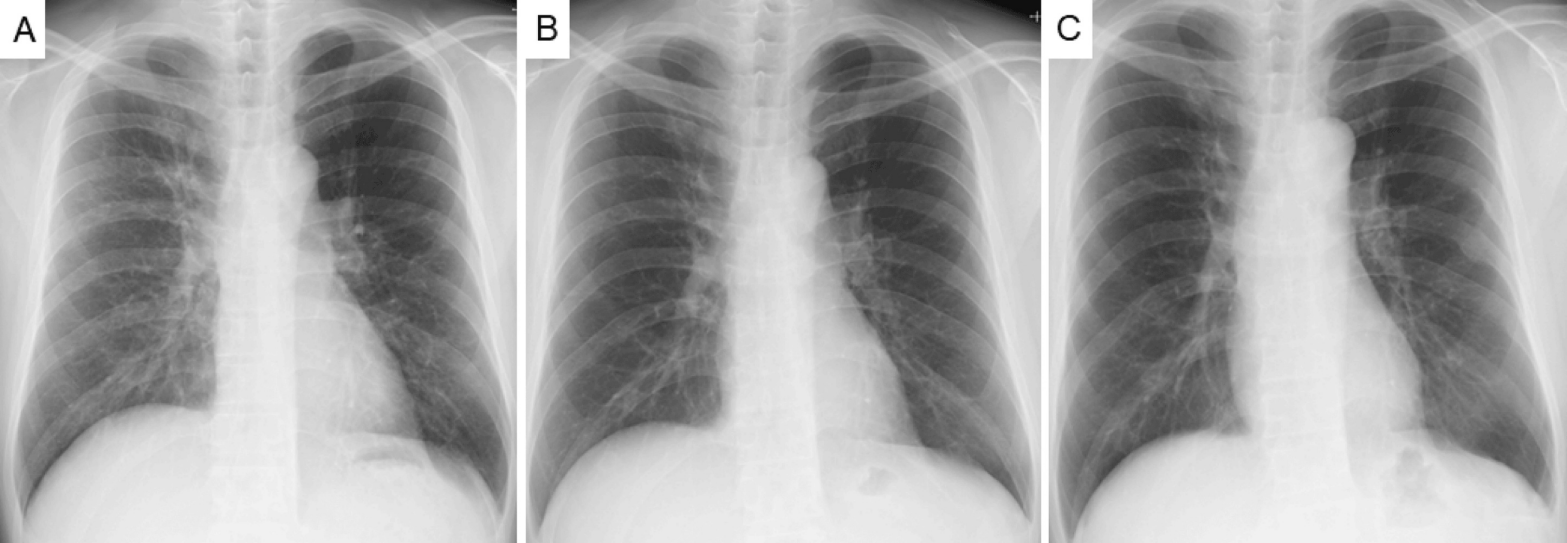
Serial chest X-rays demonstrating sustained long-term improvement after bronchoscopic mucus plug removal. (A) Chest X-ray obtained one week after bronchoscopy showing early improvement in bronchovascular bundle thickening. (B) One year later, the bronchovascular markings have nearly normalized. (C) Six years after the procedure, the findings remain stable with preserved improvement and no evidence of recurrence, consistent with the patient’s long-term symptom-free course.

Inhaled therapy for asthma (inhaled corticosteroid/long-acting β₂-agonist combination therapy) has been continued without any change before or after bronchoscopy. No recurrence of ABPM has been observed during six years of follow-up, with the patient remaining clinically stable without symptom exacerbation. Serial chest X-rays demonstrated no radiologic worsening throughout this period, confirming the absence of recurrence (Figure 5).

## Discussion

This case suggests that ABPM, which is generally treated with systemic corticosteroids or antifungal agents, may achieve complete remission through bronchoscopic removal of mucus plugs alone. The patient remained entirely free of recurrence for more than six years - far exceeding the recurrence-free intervals reported in previous studies, which have ranged only from approximately nine months to three years [2-5] - underscoring the potential long-term effectiveness of bronchoscopic deobstruction in carefully selected cases.

Previous reports suggest that the therapeutic effect of bronchoscopic mucus plug removal in ABPM arises not only from relieving mechanical airway obstruction but also from eliminating a concentrated source of fungal antigens contained within the plugs. By reducing this intrabronchial antigen load, plug removal interrupts the allergic inflammatory cascade and promotes rapid clinical improvement, even in the absence of systemic corticosteroids or antifungal therapy [6-10]. Taken together with our present case, these findings indicate that prioritizing bronchoscopic removal of mucus plugs may be a reasonable initial strategy, with pharmacologic treatment subsequently considered based on the clinical course.

Among previously reported cases of ABPM in which bronchoscopic removal of mucus plugs contributed to clinical improvement, most involved additional therapies such as inhaled bronchodilators, mucolytic agents, or inhaled corticosteroid/long-acting β₂-agonist therapy [2-5]. In fact, the only published case in which treatment remained unchanged before and after bronchoscopy is the report by Nasu et al. [4], although the follow-up period in that case was limited to approximately nine months. Moreover, even in other reported cases showing clinical improvement after bronchoscopic intervention, recurrence-free follow-up has been documented only up to approximately three years at most [2-5]. In contrast, our patient achieved complete remission through bronchoscopic mucus plug removal alone, without any change in asthma therapy and without systemic corticosteroids or antifungal agents, and has remained entirely free of recurrence for more than six years. In our case, the favorable clinical course may have been facilitated by the fact that the majority of the mucus plugs could be successfully removed during bronchoscopy, thereby achieving a substantial reduction of the intrabronchial antigen burden. This represents the longest recurrence-free period reported to date for bronchoscopy-only management of ABPM and reinforces the therapeutic relevance of bronchoscopic intervention in this condition.

Moreover, previously reported cases of ABPM successfully managed with bronchoscopic removal of mucus plugs without systemic corticosteroids or antifungal therapy have involved a limited range of causative fungi, including *Schizophyllum commune*, *Aspergillus fumigatus*, *Curvularia lunata*, *Cordyceps farinosa*, and *Curvularia mebaldsii* [6-10]. To the best of our knowledge, *Penicillium citrinum* has not been reported as a causative organism in bronchoscopy-only remission of ABPM, making the present case the first documented example. In addition, our findings provide clinical corroboration of the potential allergenic relevance of Penicillium citrinum, as previously suggested by Chen et al. [4], further underscoring the importance of this environmental fungus in allergic airway disease.

Although systemic corticosteroids remain the cornerstone of ABPM management, their long-term use carries risks such as immunosuppression, metabolic complications, and relapse upon tapering. In this context, bronchoscopic intervention offers a targeted, low-risk alternative capable of rapidly reducing airway obstruction and antigen burden. While this approach may not be suitable for all patients - particularly those with diffuse disease, uncontrolled asthma, or extensive mucus impaction - the present case highlights that carefully selected patients may achieve durable remission without pharmacologic escalation. Further accumulation of similar cases will be essential to clarify patient selection criteria and to more clearly define the role of bronchoscopic therapy within the broader treatment algorithm for ABPM.

## Conclusions

In summary, this case suggests that allergic bronchopulmonary mycosis caused by *Penicillium citrinum* may achieve complete and durable remission after bronchoscopic removal of mucus plugs alone, without systemic corticosteroids or antifungal therapy. Although systemic pharmacologic therapy remains the standard of care for ABPM, our case illustrates that bronchoscopic deobstruction may contribute to clinical remission in carefully selected patients. The patient has remained free of recurrence for more than six years, representing the longest bronchoscopy-only remission reported to date. Together with previous reports showing that mucus plug extraction reduces both airway obstruction and intrabronchial fungal antigen load, our findings suggest that bronchoscopic removal of mucus plugs may serve as a reasonable adjunctive or initial therapeutic approach in selected cases, with systemic therapy subsequently considered based on the clinical course.
